# Relationship between impulsivity, hyperactivity and working memory: a differential analysis in the rat

**DOI:** 10.1186/1744-9081-2-10

**Published:** 2006-03-28

**Authors:** Françoise Dellu-Hagedorn

**Affiliations:** 1 Laboratoire de Neuropsychobiologie des Désadaptations, CNRS UMR 5541, Université Victor Segalen Bordeaux 2 - BP. 31, 146 rue Léo Saignat; 33076 Bordeaux cedex, France

## Abstract

**Background:**

Impulsivity is a behavioural trait that comprises several distinct processes. It is a key feature of many psychopathologies such as mania, addictive disorders or attention deficit-hyperactivity disorders. To date, the aspects of impulsiveness involved in these pathologies have not yet been explicitly defined. In these disorders, sensation or drug seeking and cognitive deficits are closely related, but the nature of these relationships remains largely unknown. A new animal model of impulsiveness based on spontaneous inter-individual differences is proposed here to help clarify the relationship between characteristic aspects of impulsive-related pathologies.

**Methods:**

Rats were divided into sub-groups according to their scores in three operant tasks with varying degrees of behavioural inhibition, timing and motor vs. cognitive impulsivity demands. These tasks included a fixed consecutive number schedule (ability to complete an action to receive a reinforcer), a multiple fixed-interval/extinction schedule of reinforcement (high level of responding), and a delayed reward task (delay discounting). In addition, measurements of locomotor responses to novelty and to amphetamine in a circular corridor, and working memory in an 8-arm radial maze were obtained.

**Results:**

Substantial behavioural inter-individual differences were observed in each task, whereas few inter-task relationships were found. Impulsive rats, as defined in a task requiring inhibition of premature responses, presented a higher increase in amphetamine-induced locomotion. Reduced working memory performance was only observed in hyperactive rats in an extinction schedule.

**Conclusion:**

This novel approach shows that distinct aspects of impulsiveness and hyperactivity can be expressed based on large inter-individual differences that vary from poorly to highly adapted behaviours ones in a normal population of rats. Inhibitory deficit was related to a higher response to psychostimulants a characteristic of rats predisposed to amphetamine self-administration and related to higher limbic dopaminergic activity, whereas working memory capacity was only related to hyperactivity. This approach allows for the identification of particular individuals presenting distinct behavioural characteristics of impulsive-related psychopathologies. These individuals may be of great interest in the modelling of these disorders and the exploration of their neurobiological bases.

## Background

The concept of impulsivity covers a wide range of "actions that are poorly conceived, prematurely expressed, unduly risky, or inappropriate to the situation and that often result in undesirable consequences" [1]. The multi-factorial nature of this personality trait [2], which is well illustrated by this definition, has been largely ignored because of a lack of consensus on its definition and appropriate measures. Impulsivity is mentioned in the DSM-IV diagnostic criteria as prominent in several psychiatric disorders, but it is never explicitly defined [3]. It is a key aspect of mania, personality disorders and conduct disorders, and is also regarded as the most relevant symptom in attention deficit-hyperactivity disorder (ADHD) [4-6]. It also plays a key role in substance abuse [7,8], a disorder clearly associated with conduct disorders [9,10] as well as ADHD [11]. Furthermore, personality theorists have identified a factor called impulsive unsocialized sensation-seeking, linking impulsivity to the sensation-seeking trait. This factor has been shown to correlate with early onset of drug use and later drug abuse [12-15].

Impulsivity-related psychiatric disorders are also characterized by deficits in executive functioning, notably in working memory [16]. These deficits have been demonstrated in personality and conduct disorders [17-19], ADHD (for review, see [20]), mania [21-24], and subjects predisposed to substance abuse [25-29]. The associations of these psychiatric disorders with impulsivity and executive function deficits suggest that common brain mechanisms may underlie their aetiologies.

An animal model revealing the relationship between impulsivity and the related behaviours described above would provide valuable insight into these disease states. Recently, impulsivity has been studied more systemically in animals by devising various operant behaviour tasks revealing its non-unitary nature [30]. However, information relating to the inter-relationship between characteristics of impulsivity [31] are just beginning to emerge.

To address this issue, a new animal model incorporating different symptoms of the psychopathologies related to impulsiveness was investigated. The primary objective in developing this model, based on inter-individual variability in behavioural responses, was to determine to what extent various aspects of behaviours *that could be related to *impulsiveness can be spontaneously expressed in a normal population of rats. Impulsiveness and hyperactivity were tested in tasks covering different aspects of cognitive vs. motor impulsive behaviours that have all been clinically related to impulsivity. They involved various degrees of timing and behavioural inhibition demands: a fixed consecutive number schedule (FCN8) that measured the rat's ability to terminate an action to reach a goal, a multiple fixed-interval/extinction schedule of reinforcement (FI EXT) to measure responding during waiting periods, and a test assessing delay discounting where a choice between a small immediate reward, or a bigger one after a waiting period was measured. The last two tasks could reveal large deficits (in very similar paradigms) in impulsive-related psychopathologies (ADHD) [32,33]. Furthermore, by measuring these behaviours in the same individuals, it was possible to determine the nature of the relationship between the behaviours.

The second aim in developing this new model was to identify which aspects of these behaviours were related to novelty seeking, the response to psychostimulants, and/or to cognitive deficits. It has been reported that measures of sensation-seeking are highly correlated with novelty preference in humans [34]. Similarly, the locomotor response to novelty in a circular corridor has been shown to predict novelty-seeking in rats [35,36], a trait that has been linked to increased responsiveness to psychomotor stimulants and a predisposition to drug self-administration [37]. Important individual differences in working memory have been reported in normal rats in an 8-arm radial maze [38], but the relationship between working memory capacity and impulsivity in animals is yet to be investigated. Identifying a relationship between novelty and response to a drug challenge and cognitive capacity was achieved by comparing the performance of rats selected for their scores in the different behavioural measures.

Substantial inter-individual differences in behavioural responses were observed in each task, while few inter-task relationships were found. Inhibitory deficit was related to a higher response to psychostimulants, whereas working memory capacity was only related to an extinction deficit. Delay discounting was found unrelated to any other behavioural measures.

## Methods

### Animals

Forty male Sprague-Dawley rats (Charles River, Lyon, France) were received at six weeks of age. They were housed in groups of four in a temperature (22°C) and humidity controlled room (60%) on an inverted 12 h light-dark (8:00–-20:00) schedule. They had free access to food and water except during the impulsivity and working memory testing periods where animals were under dietary restriction. Food rationing was adjusted in order to maintain their weight between 80% and 85% of their expected weight at the same age. A week before the beginning of the experiments, animals were handled for a few minutes every day.

### Apparatus and behavioural testing

All experiments were performed in accordance with the European Communities Council Directive of November 24, 1986 (86/609/EEC). They were carried out with respect of the inverted nyctemeral cycle.

### Impulsive-related behaviours

Several behavioral responses were assessed using three different experimental protocols and the principal characteristics are summarized in Table 1. The main distinction between these tasks is between cognitive vs. motor impulsiveness. Impulsive behaviour that has negative consequences or that leads to a lower efficiency is related to poorly conceived or prematurely expressed actions, and could therefore be referred to as cognitive impulsivity. It concerns failure to resist an impulse, drive or temptation and acting without consideration of alternatives and/or consequences. Motor impulsivity could rather refer to a higher level of activity which is not adequate to the environmental contingency, i.e. in a situation that does not require activity (i.e. a waiting situation), with no direct negative consequences.

**Table 1:** Principal characteristics of the tasks measuring impulsive-related behaviours.

*Tasks*	Cognitive impulsivity		Motor impulsivity	
	
*Requirements*	Choice	FCN8	FI	EXT
Evaluation of negative consequences	+++	+++	0	0
Behavioral inhibition	0	+++	+++	+++
Waiting, tolerance to delay of gratification	+++	0	+++	+++
Timing to avoid inefficient responses	0	+++	+++	0

Cognitive impulsiveness was assessed by two different tasks: a delay discounting task (choice), which assess mainly the ability to wait and the tolerance to delay of gratification and a fixed consecutive number schedule of reinforcement (FCN8), measuring the ability to terminate an action to reach a goal. This task requires both timing and inhibition of premature responding and does not require waiting contrarily to the previous one. Motor impulsivity was tested through two different schedules measured in a two-component 2-min fixed interval (FI) 5-min extinction (EXT) schedule of reinforcement. During these tasks, intolerance to a waiting period is also indirectly assessed through behavioural disinhibition. Only the FI component requires timing and measures anticipation of food reward delivery.

The configuration of the apparatus and the order of each task (Fixed consecutive number schedule, multiple fixed-interval/extinction schedules of reinforcement and delay-discounting task successively), were chosen to minimize any possible interference between protocols (see discussion). Thirty minutes before a session, rats were placed in their home cage, in a light-attenuated experimental room.

The apparatus consisted of eight sound-insulated light-tight outer chambers each containing a two lever conditioning box (Imetronic, Pessac, France), as previously described [39].

#### Fixed consecutive number schedule (FCN8)

This task, adapted from [40], measures that ability of the rat to carry out a chain of sequential acts in order to achieve a goal. The schedule required a fixed minimum number of 8 responses on one of the levers, before a response on the second lever resulted in food delivery. A reduction in the average chain length may be the sign of a loss of behavioural inhibition.

On the first day, the levers were retracted and the rats were placed in the operant chambers for 30 min with 10 food pellets placed in the food tray. On day 2, they were tested under a fixed time schedule of reinforcement in which one food pellet was delivered every 60s in a non-contingent manner for 30 min. On day 3 of training, the left lever was inserted into the box, and every press resulted in the delivery of a food pellet. On the following day, the right lever was inserted and the same schedule of reinforcement was employed. This alternation procedure was continued until the rats had pressed both levers at least 100 times in less than 20 min.

Fixed consecutive number training was then begun. On the first day the rats were required first to press the left lever (FCN lever) and then the right lever (reinforced lever) to obtain food (FCN1) during a 45-min session. This session was continued until the rats had obtained at least 60 pellets. Then, the FCN requirement was increased to 2 and according to the same criterion, to 3, 5 and 8 (test condition). If the chain was shorter than 2, 3, 5 or 8 (respectively), the rat was required to start a new chain. If the chain was longer, it had no consequence and the pellet was delivered when the rat pressed the reinforced lever. Rats that failed to reach the criterion to be tested under a FCN8 schedule after 20 training sessions were excluded. The other rats were tested under the FCN8 schedule for 8 days. The mean scores of each animal obtained from day 2 to 8 were recorded.

The following parameters were recorded: mean length of the chain of responses made on the FCN lever before switching to the reinforced lever; percentage of efficient chains of presses; proportion of total number of chains long of *n *consecutive presses; mean total number of both lever responses per min; latency to food pellet collection and number of sessions needed to reach the test phase (learning score).

#### Multiple fixed-interval/extinction schedules of reinforcement

In this experiment, a fixed-interval schedule of reinforcement operates alternately with an extinction component (adapted from [41]). For a fixed-interval schedule, responses during the time interval have no consequence, but the first response after the interval has elapsed produces the delivery of the reinforcer. According to Sagvolden [32], the fixed-interval component measures reactivity to reinforcers, activity and motor impulsiveness and the extinction component measures sensitivity to stimulus change and sustained attention.

In this protocol, only the right lever was available. This lever had previously been the less used lever (reinforced lever) in the FCN8 schedule. During the fixed-interval component (FI), the house light was on and the first lever press after a time-interval was reinforced by a pellet. A light above the lever was on when the pellet was available and was off when the rat visited the tray. During the extinction component (EXT, 5 min), there was no house light and no pellet was delivered. During each session, the FI and EXT components operated twice in alternation. Rats were first trained with 5 sessions with a multiple 30s FI-EXT schedule. Then, rats were trained with two sessions with a multiple 1 min FI-EXT schedule followed by sessions with the final multiple 2 min FI-EXT schedule test conditions. A maximum of 7 pellets per FI was delivered during the last two conditions. Six test sessions were necessary to obtain stabilisation of performances for all rats and the means of the next 5 sessions were used for statistical analysis.

The 2 min FI component was divided into 12 consecutive 10s segments and the 5 min EXT component was divided into five consecutive 1 min segments. The mean number of lever presses was recorded as a function of FI and EXT segments. As described earlier, data from the initial FI after the start of the session, as well as that from the first interval following the first EXT were excluded because the behaviour during these intervals might deviate from that during the other intervals [42]. The number of pellets delivered and the number of visits to the empty tray as well as speed in collecting pellets were also measured for FI and/or EXT components.

#### Delay-discounting

The protocol of delay-discounting measures intolerance to situations when the reward is delayed, through the preference of a smaller immediate reinforcer to larger rewards, which come only after a delay (adapted from [43,44]). By increasing the delay to reinforcement, the relationship between the magnitude of delay and choice between larger, delayed or smaller, immediate reinforcers could be determined. In this way, it was possible to assess the devaluation of the large reward as a function of time and the delay at which the smaller reward is perceived to be of greater value for each individual.

Rats were trained over at least 5 daily sessions in the conditioning box with two levers available. During the initial phase, a press on the left lever (L1) resulted in delivery of one food pellet (45 mg, Bioserv, USA) whereas a press on the right lever (L5) delivered five pellets. Whenever a reinforced lever press was made, a light above this lever was switched on for 1s. Three seconds after food delivery, the magazine light was turned on for 25s, during which time additional presses were without consequences (time-out). The end of this time-out was signalled by a 1s light extinction. Given that an additional lever (left one or L1) was added compared to the previous protocol (FI/EXT schedule), a training period was undergone in order to obtain stable performances with no interference with previous requirements. This training period was continued until the rats made more than 70% L5 selections with less than 15% variation in this score on 2 consecutive sessions (5 to 10 sessions were necessary).

During the test, a delay was inserted between pressing L5 and the delivery of the five pellets. During this delay, the light above the right lever remained on until the pellet was eaten and thus could signal the waiting period. The same delay was inserted after the immediate delivery of food following a press on L1. The delay was fixed for a given daily session and increased progressively over the days by 5s from 0 to 30s according to a criterion of stabilization before increasing the delay: scores over two consecutive sessions should not vary by more than 10%. All sessions ended when 100 pellets had been delivered.

Percentage of L5 choice, number of sessions necessary to reach the criterion, number of visits to the empty tray, total number of presses, and presses during the time-out periods were measured. These parameters were calculated for each delay as the mean of the last two stabilized sessions.

### Working memory

Animals started training a week after the last impulsivity test, in a different experimental room. The 8-arm radial maze and behavioural procedure have previously been described in detail [39]. Briefly, working memory was measured by the ability to visit the eight baited arms without re-entry [45], during daily trials over 6 days. The trial ended when the rat had visited each of the 8 arms or after 16 visits.

Total number of errors, number of errors during the 8-first choices and mean time taken to reach the pellet after opening of the doors were analysed.

### Novelty and amphetamine-induced locomotor activities, basal nocturnal activity

The novel environment consisted of a circular corridor (10 cm wide, 70 cm in diameter) equipped with four photoelectric cells placed on the perpendicular axes automatically recording locomotion (Imetronic, Pessac, France) outside the testing room.

Rats were tested two weeks after being fed ad libitum. Initial locomotor response was recorded over a period of 2 h (4 pm. – 6 pm.) and total number of photocell counts reflected reactivity to novelty. At 6 pm., a bottle of water and a food dispenser were suspended in the middle of the corridor. Light was turned off from 8 pm. to 8 am and basal nocturnal activity was then measured over 11 hours, from 9 pm to 8 am. A week later, at 2 pm, the animals were placed in the same corridors for 2 h. Then, locomotor response to vehicle (1 ml/kg i.p.) was measured during 30 min followed by locomotor response to amphetamine (1 mg/kg/i.p.) recorded over 2.5 h every 10 min (16.30 h–19.00 h).

### Analysis of individual differences in impulsive-related behaviours

In this study, a dimensional approach based on a correlational study was used to assess the relationships between the different processes. It was combined with a typological approach that consisted, after selecting the most representative parameter measured in a given task, in extracting subgroups of individuals with low (LOW) vs high (HIGH) scores according to the upper and lower quartiles in each task, the remainder constituting an intermediate group (INT). Their behaviours were then compared during the same or distinct tasks. It is possible for a rat from an extreme quartile to have identical scores to the following intermediate animals. In that case, it was included in this latter group. This method was used to describe extreme behaviours in each task, given these behaviours are described with a time-course or according to the length of a waiting period.

### Statistical analysis

Comparisons of scores between groups were made using analysis of variance (ANOVA), followed by analysis of simple main effects *(SME) *and by *post-hoc *comparisons using the Newman-Keuls *(NK) *test, when appropriate. Student's *t*-tests were used to compare scores of the different groups or assess departure from chance. Correlations between scores were evaluated using Bravais-Pearson's correlation test. General Linear Model (GLM) was used to assess the relationships between impulsivity parameters and working memory scores as well as locomotor response to amphetamine. P-values of multiple comparisons analysis made on continuous or discontinuous (classifications) variables of impulsivity were adjusted using the False Discovering Rate (FDR) controlling procedures [46].

The normality of the variable distribution was verified using Shapiro-Wilk's test. A logarithmic transformation was performed when necessary to normalize variables.

## Results

### Individual differences in impulsive-related behaviours

#### Fixed consecutive number schedule (FCN8)

Four rats were excluded from this analysis because they did not reach the criterion for the test phase. They needed significantly more sessions to reach the criterion on the FCN3 schedule (10.7 ± 1.0) compared to the others (4.6 ± 0.3) (*t *= 6.06; *df *= 38, *p *< .001).

**Figure 1:**
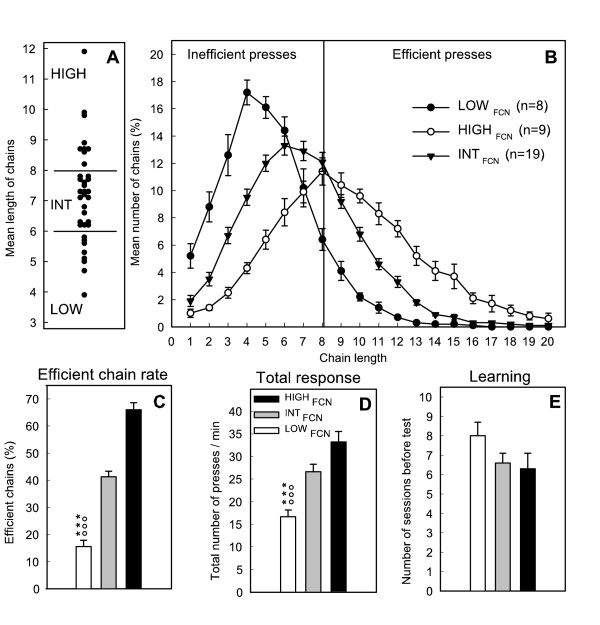
Inter-individual differences in impulsivity of rats measured in the FCN8 task. A: distribution of individual scores of rats (mean chain length over the last 7 days) and selection of rats with high level of inhibition (HIGH_FCN_, scores > 8) low level of inhibition (LOW_FCN_, scores < 6) and intermediate levels (INT_FCN_); B: distribution (%) of mean chain lengths of efficient (≥ 8) or inefficient chains (< 8) of the three groups. Optimal performance (8) is indicated by the vertical line. C: percentage mean of efficient chains; D: total number of presses per min and E: number of sessions needed to reach the test phase of the three groups. ANOVA (NK): ***, *p *< .001 for comparisons between LOW and HIGH groups; °°°, *p *< .001 for comparisons between LOW and INT groups.

The mean chain length and percentage of efficient chains (chains ≥ 8) as well as response rate were positively correlated. The mean chain length, ranging from 3.9 to 11.9 presses, was chosen, (as described previously [39]), to classify rats into three groups: a first group in the upper quartile defining rats with low level of inhibition (LOW_FCN_, n = 8) contained all the rats with a mean chain length below 6, a second group in the lower quartile, defining rats with high level of inhibition (HIGH_FCN_, n = 9) with a mean chain length above 8, and the third contained the remainder defined as intermediate rats (INT_FCN_, n = 19) (Figure 1).

The mean length of chains of LOW_FCN _rats was 5.1 ± 0.2, INT_FCN _rats, 7.0 ± 0.1 and HIGH_FCN _rats, 9.2 ± 0.3. The rate of responding was higher in this latter group. Learning performance (reflected by the number of sessions needed to reach the test phase) and mean latency to collect earned food pellets did not differ significantly between groups (*F*(2,33) = 1.35 and 0.91, ns).

#### Multiple fixed-interval/extinction schedules of reinforcement (FI EXT)

The number of lever presses during FI and EXT, which both followed a logarithmic distribution, were positively correlated. Presses during EXT were positively correlated with visits to the empty tray. No significant correlation was found between the number of lever presses during FI or EXT and speed in collecting food. The mean number of lever presses ranged from 21 to 258 during FI and from 2 to 107 during EXT. A positive correlation was shown between the number of presses during FI and EXT, but an examination of individuals revealed that half of the 10 most active rats during FI were very inactive during this period. These two parameters were therefore chosen to classify rats into groups.

#### Presses during FI

**Figure 2:**
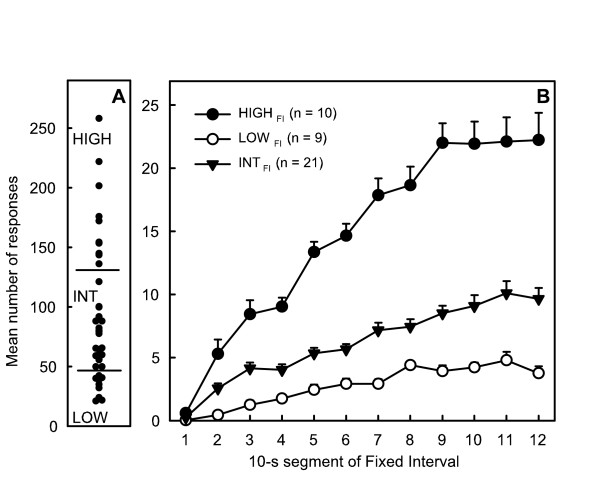
Inter-individual differences in activity of rats measured during the FI schedule: A: distribution of individual scores of rats (mean number of presses over the last 5 sessions) and selection of hypoactive (LOW_FI_, scores < 50) hyperactive (HIGH_FI_, score > 130) and intermediate rats (INT_FI_); B: mean number of lever presses by each group during the 2-min FI component as a function of 10-s segments of the FI period.

Selection of rats with hyperactivity (HIGH_FI_, n = 9), hypoactivity (LOW_FI_, n = 10) and intermediate group (INT_FI_, n = 21) according to the mean number of lever presses during FI and time-course of their activity are represented in figure 2. HIGH_FI _activity was 3 and 6 times higher than that of the INT_FI _and LOW_FI_, respectively. It was particularly pronounced at the end of every FI, reaching a plateau 40s before the end of FI. The mean speed in collecting reinforcers did not differ significantly between groups as did the number of visits in the empty tray (*F*(2,37) = 1.35 and 0.32, ns).

#### Presses during EXT

**Figure 3:**
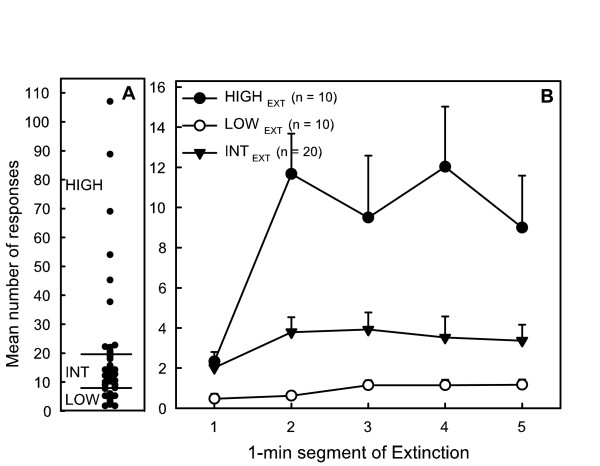
Inter-individual differences in activity of rats measured during the EXT schedule: A: distribution of individual scores of rats (mean number of presses over the last 5 sessions) and selection of hypoactive (LOW_EXT_, scores < 8) hyperactive (HIGH_EXT_, scores > 20) and intermediate rats (INT_FI_). B: Mean number of lever presses by each group during the 5 min EXT component as a function of 1 min segments of the EXT period.

Selection of rats with hyperactivity (HIGH_EXT_, n = 10), hypoactivity (LOW_EXT_, n = 10) and intermediate group (INT_EXT_, n = 20) according to the mean number of lever presses during EXT and time-course of their activity are represented in figure 3. HIGH_EXT _activity was about 4 and 10 times higher than that of INT_EXT _and LOW_EXT_, respectively. It significantly increased with time (*F*(4,148) = 25.38, *p *< .001) but remained stable at a low level for LOW_EXT _and INT_EXT_(*F*(4,148) = 0.18 and 0.99 respectively, ns).

#### Delay-Discounting: Choice between an immediate small reward or a delayed bigger reward

As expected, following the training period, animals significantly preferred the L5 lever delivering the large reward (L5 choice: 88.9% ± 1.5). The preference progressively shifted towards L1 as the delay increased. A large increase in visits to the empty tray was observed when the delay was increased, whereas the number of lever presses during the time-out periods significantly increased during the longer delays (25–30 s). No significant correlation was observed between mean percentage of L5 choice and the number of visits to the empty tray or with the number of presses during the time-out periods. These last two variables were negatively correlated. Total activity was positively correlated with activity during time-out periods and negatively correlated with visits to the empty tray. No correlation was found between L5 choice and total number of sessions required to reach each step of the test.

**Figure 4:**
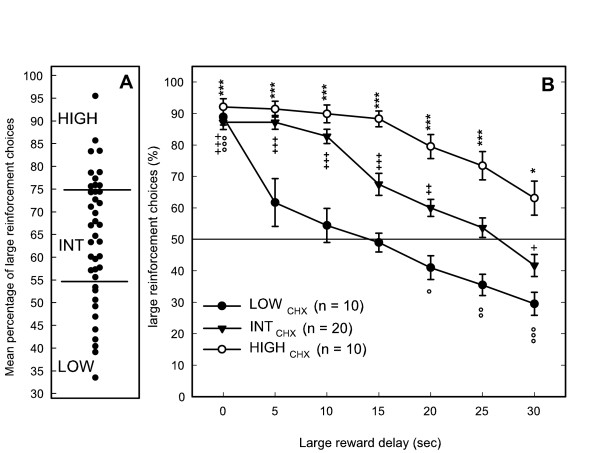
Inter-individual differences in impulsivity measured in the delay-discounting task: A: distribution of individual scores of each rat (mean percentage of choice for the large reward after 5 to 30 min delays) and selection of rats with high choice for large reward (HIGH_CHX_, scores > 75%) low choice for large reward (LOW_CHX_, scores < 55%) and intermediate rats (INT_CHX_). B: Percentage choice of the large reinforcement of the three groups according to the length of the delay before obtaining it: LOW_CHX _had no preference for any lever at delays 5 to 15 min and then shifted their preference for the immediate reward, whereas HIGH_CHX _preferred the largest reward whatever the delay. Comparisons with chance level (50%): significant difference with chance for scores above or below 50% show a preference for the large or the small reward respectively. Student *t*-test: ***, +++, °°°, *p *< .001; **, ++, °°, *p *< .01; *, +, °, *p *< .05.

Important individual differences were observed in the time lapse at which rats no longer preferred pressing L5 (breakpoint): as early as the 5 sec delay for 4 rats whereas 10 rats still preferred to press more on L1 during the 30 s delay. The mean percentage of L5 choice during delays lasting between 5 and 30 s was chosen to classify rats with low choice for large reward (LOW_CHX_, n = 10), high choice for large reward (HIGH_CHX_, n = 10) and intermediate (INT_CHX_, n = 20) (Figure 4). All groups showed a marked preference for L5 when it was delivered with no delay and their score did not differ (88.9% ± 2.6; 87.2% ± 2.3 and 92.1% ± 2.6 respectively). As the delay increased, their choices significantly differed (*F*(2,37) = 66.64, *p *< .001) with a different time-course (*F*(12,222) = 4.07, *p *< .001) (Figure 4). The three groups showed no difference in the number of lever presses during the time-out periods or in the number of visits to the empty tray (*F*(2,37) = 0.5 and 1.34, ns). The total number of sessions required to reach each step of the test was not significantly different between groups (*F*(2,37) = 2.77, ns). Two of the LOW_CHX _and two of the INT_CHX _were rats previously excluded from the fixed consecutive number schedule task.

### Relationships between scores in the different impulsivity tasks

Statistical results of correlation analysis between scores obtained in the different tasks of impulsivity are summarized in table 2. Whereas several of the variables measured within the same task were correlated, only a few inter-task correlations were found. No significant correlation could be shown between choice of a delayed reward and any other parameters. However, lever presses during the time-out periods in this task were positively correlated with total lever presses during the FCN task and during FI in the multiple fixed-interval/extinction schedules of reinforcement task. Positive correlations were also found between total number of lever presses during FI and EXT and total number of presses during the delay-discounting task.

**Table 2:** Correlations within and between different measures of impulsive-related behaviours.

			FCN 8		FI-EXT			CHOICE			
			
			CHLENGHT	TOT ACT	FI ACT	EXT ACT	VET	CHOICE	VET	TOUT	TOT ACT
FCN 8	mean chain lenght	CHLENGHT	-	0.62 ***	-0.05	-0.36	0.12	0.04	0.09	0.25	0.09
	total activity	TOT ACT		-	-0.03	-0.20	0.08	0.07	-0.09	0.45 *	0.03
FI-EXT	activity during FI	FI ACT			-	0.48 *	0.16	-0.04	-0.31	0.50 ***	0.56 **
	activity during EXT	EXT ACT				-	0.33	-0.08	-0.12	-0.27	0.44 *
	visits in empty tray	VET					-	0.10	-0.05	0.18	0.25
CHOICE	mean % choice for larger reward	CHOICE						-	-0.22	-0.23	0.05
	visits in empty tray	VET							-	-0.39 *	-0.36
	activity during time out	TOUT								-	0.39 *
	total activity	TOT ACT									-

Bravais-Pearson's correlation test values (*r*); ***, *p *< .001; **, *p *< .01; * *p *< .05 (adjusted *p*-values with FDR procedure).

Mean chain length of chains measured in the FCN8 test was negatively correlated with activity during EXT in the multiple fixed-interval/extinction schedules of the reinforcement task.

Analyses of comparisons between extreme subgroups could better illustrate the relationships between impulsivity tasks and reveal some differences that give additional information. The main statistical comparisons between groups are summarized in table 3.

**Table 3:** Comparisons of subgroups for different impulsive-related behaviours.

		FCN 8	FI	EXT	CHOICE
		
		LOW _FCN _*vs *HIGH _FCN_	HIGH _FI _*vs *LOW _FI_	HIGH_EXT _*vs *LOW _EXT_	LOW _CHX _*vs *HIGH _CHX_
FCN 8	chain lenght		ns	HIGH_**EXT **_< LOW_EXT_**	ns
	total activity	LOW _FCN _< HIGH _FCN _***	ns	ns	ns
FI-EXT	activity during FI	ns		HIGH _EXT _> LOW _EXT _**	ns
	activity during EXT	ns	ns		ns
CHOICE	% choice for larger reward	ns	ns	ns	
	activity during time out	ns	HIGH_**FI **_> LOW_FI_***	ns	ns
	total activity	ns	HIGH_**FI **_> LOW_FI_**	HIGH_**EXT **_> LOW_EXT_**	ns

Statistical comparisons between scores obtained across tasks of rats selected for their level of inhibition in the Fixed Consecutive Number schedule of reinforcement task (FCN8); their level of activity in the Multiple Fixed interval/extinction schedules or their choice for the larger reward in the Delay discouting task. Student *t *test: ***, *p *< .001; **, *p *< .01 ; * *p *< .05; ns, non significant.

**Figure 5:**
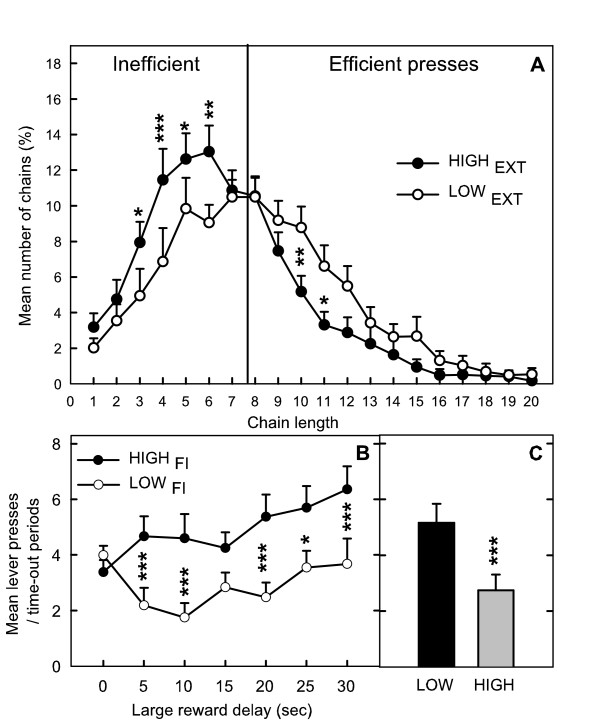
Relationships between scores in the different impulsivity tasks. A: distribution of efficient and inefficient chains (%) in the FCN8 task of hyperactive (HIGH_EXT_) and hypoactive rats (LOW_EXT_) selected in the EXT schedule. B: choice of the large reinforcement (%) in the delay-discounting task according to the length of the delay before obtaining it for hyperactive (HIGH_FI_) and hypoactive (LOW_FI_) rats selected in the FI schedule. C: mean percentage of choice for the big reward at delays 5 to 30 min. ANOVA (NK): ***, *p *< .001; **, *p *< 0.01; *, *p *< 0.5.

Correlations between variables obtained in the different tasks were not systematically corroborated by comparisons between subgroups. When groups were selected from EXT, they significantly differed in the impulsivity score obtained in the FCN8 task (mean chain length). HIGH_EXT _is of particular interest: these rats also obtained shorter chains of lever presses in the FCN task, whereas LOW_EXT _show intermediate scores of impulsivity in this task (ANOVA group × chain length: *F*(19,304) = 2.39, *p *< .001) (Figure 5). This relationship seems to be related to these particular HIGH_EXT _given that LOW_FCN _does not exhibit significantly higher activity during EXT compared to HIGH_FCN _(Table 3).

The relationship between measures of motor impulsivity during FI and measures of activity in the delay discounting task reveals that two independent kinds of behaviour can be identified in the latter task. Whereas LOW_CHX _and HIGH_CHX_, selected in this task, did not differ in the lever presses during the time-out periods (*F*(2,37) = 0.44, ns), HIGH_FI _were significantly more active than LOW_FI _during these periods (*F*(2,38) = 8.12; *p *< .001, NK, LOW vs HIGH, *p *< .001) (Figure 5).

### Relationships between impulsive-related behaviours, response to novelty and amphetamine-induced locomotor activity

Overall score of locomotor reactivity to the novel environment over two hours was 450 ± 22 photocell counts, the lower value being 187 and the highest, 782. Locomotor reactivity returned to baseline one hour after the beginning of the experiment. Locomotor reactivity to saline injection lasted 10 min and activity then returned to baseline. The effect of amphetamine was then tested and overall scores of locomotor activity over 150 min were 603 ± 53 photocell counts, the lower value being 124, the highest 1510.

Positive correlations were found between scores of reactivity to novelty and nocturnal activity (*r *= 0.35, *df *= 38, *p *< .05) as well as response to amphetamine (*r *= 0.49, *df *= 38, *p *< .001). Response to amphetamine was not correlated with nocturnal activity (*r *= 0.07, *df *= 38, ns).

None of the impulsivity tasks provided support for a link between impulsiveness and locomotor response to novelty or with basal nocturnal locomotor activity. However, evidence was found for a relationship between locomotor response to amphetamine and impulsivity in the FCN8 test. Results are summarized on Table 4.

**Table 4:** Summary of the results of locomotor activities of the different subgroups.

Impulsive task	groups	Novelty-induced locomotor activity	Basal nocturnal locomotor activity	Amphetamine- induced locomotor activity
Fixed consecutive number schedule (FCN 8)	LOW_FCN_	365.5 ± 49.7	614.0 ± 78.2	252.2 ± 32.3 **
	INT_FCN_	484.2 ± 27.4	595.6 ± 41.9	215.4 ± 22.9 *
	HIGH_FCN_	416.0 ± 37.9	606.1 ± 61.1	**124.2 ± 15.6**
Multiple Fixed interval/extinction schedules of reinforcement	LOW_FI_	484.6 ± 44.1	606.6 ± 49.3	216.4 ± 37.4
	INT_FI_	421.9 ± 25.6	597.7 ± 48.6	191.3 ± 21.8
	HIGH_FI_	474.3 ± 56.9	553.8 ± 48.4	188.2 ± 31.1
	LOW_EXT_	405.2 ± 45.2	551.9 ± 60.2	227.7 ± 52.2
	INT_EXT_	442.6 ± 25.8	612.3 ± 50.7	286.2 ± 28.0
	HIGH_EXT_	516.4 ± 51.6	583.6 ± 26.7	201.4 ± 37.4
Delay discounting	LOW_CHX_	418.8 ± 48.4	517.3 ± 50.4	218.5 ± 44.7
	INT_CHX_	471.6 ± 31.1	597.2 ± 40.5	279.8 ± 33.9
	HIGH_CHX_	436.9 ± 35.2	648.4 ± 71.0	223.2 ± 28.6

Groups of rats were selected for their level of inhibition in the Fixed Consecutive Number schedule of reinforcement task (FCN8); their level of activity in the Multiple Fixed interval/extinction schedules or their choice for the larger reward in the Delay discouting task. Groups with low scores (LOW), intermediate (INT) and high scores (HIGH) in each task, were compared for their locomotor activities (mean ± SEM) in a circular corridor in three different conditions: during response to novelty (first two hours), after habituation during nocturnal phase (10 h), and 10 to 40 min after 1 mg i.p. amphetamine injection (peak of response). Statistical analysis of comparisons with HIGH, GLM with adjusted *p*-values (FDR procedure), **, *p *< .01; * *p *< .05.

**Figure 6:**
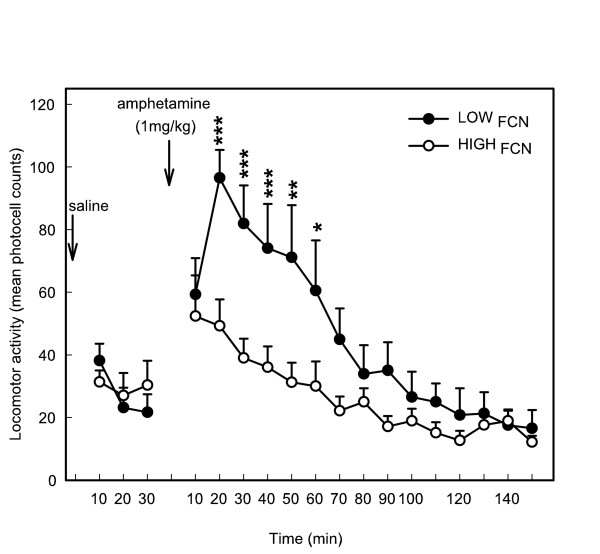
Comparisons of locomotor activity of rats with low (LOW_FCN_) and high levels of inhibition (HIGH_FCN_) selected in the FCN task in response to saline and amphetamine injections (1 mg/kg i.p.). LOW_FCN _had a higher locomotor response to amphetamine compared to HIGH_FCN_lasting 1 h after injection. LOW_FCN _locomotor activity returned to basal levels 70 min after amphetamine injection whereas it only returned to baseline after 40 min for HIGH_FCN_. ANOVA (NK): ***, *p *< .001; ** *p *< .01; * *p *< .05.

Amphetamine injection produced a higher increase in locomotor activity of LOW_FCN _compared to HIGH_FCN_, the main differences being observed at the peak of the response i.e. 10 to 40 min after the injection (*F*(2,33) = 5.02; *p *< .05; *NK*, *p *< .01, after adjusting p-values using FDR controlling procedures for multiple comparisons) (Figure 6). This response was negatively correlated with impulsivity in the FCN8 task (*r *= -0.39; *p *< .02). The time-course of this effect also differed: HIGH_FCN _activity returned faster to basal levels (interaction group × time: *F*(14,210) = 3.27, *p *< .001). No difference in activity was observed between these groups after a saline injection (*F*(1,15) = 0.06, ns). GLM performed on the continuous variable of impulsivity and the peak of response of amphetamine showed a nearly significant effect (F(1,34) = 6.07; *p *= 0.07 after adjusting p-values using FDR controlling procedures).

### Relationships between impulsive-related behaviours and working memory

**Figure 7:**
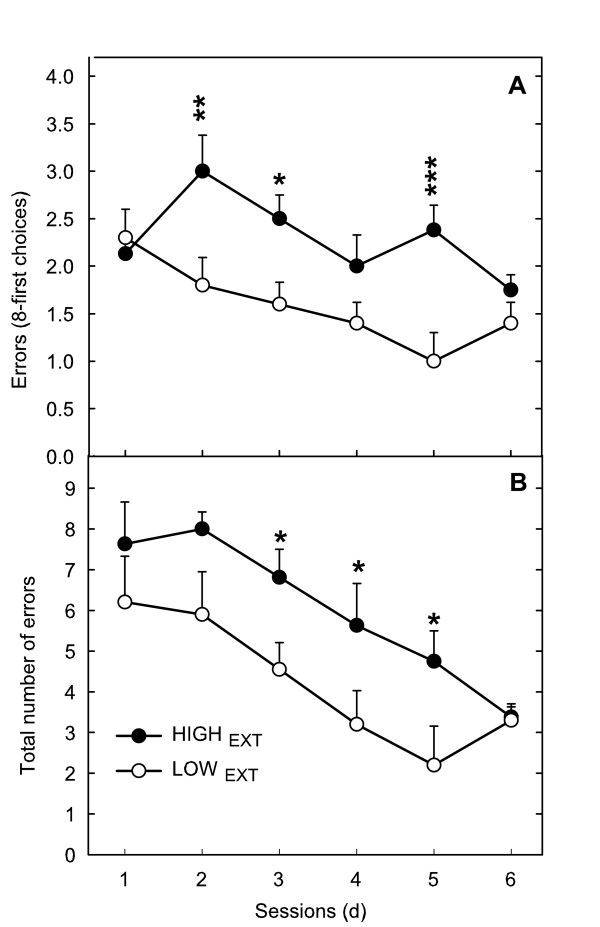
Comparisons of the time-course of working memory scores (number of errors during the eight-first choices (A) and total number of errors (B)) measured in an 8-arm radial maze of hyperactive *vs *hypoactive rats selected in the EXT schedule. HIGH_EXT _made significantly more errors compared to LOW_EXT_. ANOVA (NK): ***, <.001**, *p *< .01; * *p *< .05).

After habituation, one rat did not move in the radial maze and was highly reactive; it was therefore eliminated from this experiment. Large inter-individual differences were observed in working memory capacities: total number of errors varied between 0.8 and 7.4. Evidence was found for a relationship between working memory capacities and hyperactivity: HIGH_EXT _rats made more errors in the eight first choices as well as more total errors in the radial maze than did LOW_EXT _(*F*(1,17) = 14.28, *p *< 0.01 and 7.71, *p *= 0.05 respectively, after adjusting *p*-values using FDR controlling procedures) (Figure 7). Both groups significantly improved their scores with practice and no difference was observed between groups on the last day. The mean time to reach the end of an arm did not significantly differ between groups (*F*(1,16) = 0.04, *ns*). Activity during extinction was positively correlated with mean number of errors in the eight first choices as well as more total errors in the radial maze (*r *= 0.50; *df *= 39, *p *< .001 and *r *= 0.40; *df *= 39, *p *< .01 respectively). GLM performed on the continuous variable of activity and scores in the radial maze showed a significant relationship with the number of errors during the 8-first choices (*F*(1,37) = 8.62, *p *< .05) and a nearly significant relationship with total number of errors (*F*(1,137) = 6.47; *p *< .06) both after adjusting *p*-values using FDR controlling procedures. None of the scores measured in the other conditions could be related to working memory capacities (Table 5).

**Table 5:** Summary of working memory scores of groups of rats selected in each task.

Impulsive task	groups	Total number of errors	Errors in the first 8 choices	Time to reach the pellets
Fixed consecutive number schedule (FCN 8)	LOW_FCN_	5.4 ± 0.4	2.0 ± 0.2	8.9 ± 0.8
	INT_FCN_	4.1 ± 0.7	1.6 ± 0.2	9.7 ± 0.9
	HIGH_FCN_	4.9 ± 0.6	1.8 ± 0.1	9.0 ± 0.8
Multiple Fixed interval/extinction schedules of reinforcement	LOW_FI_	4.8 ± 0.7	1.6 ± 0.1	9.9 ± 1.2
	INT_FI_	4.6 ± 0.4	1.8 ± 0.1	9.3 ± 0.6
	HIGH_FI_	4.8 ± 0.7	2.0 ± 0.2	8.6 ± 0.6
	LOW_EXT_	**3.8 ± 0.5**	**1.4 ± 0.2**	8.7 ± 0.8
	INT_EXT_	**4.4 ± 0.4**	**1.7 ± 0.1**	8.7 ± 0.5
	HIGH_EXT_	5.7 ± 0.5*	2.3 ± 0.2**	8.3 ± 0.5
Delay discounting	LOW_CHX_	4.7 ± 0.6	1.9 ± 0.2	8.9 ± 0.5
	INT_CHX_	4.7 ± 0.4	1.9 ± 0.9	8.9 ± 0.7
	HIGH_CHX_	4.1 ± 0.7	1.5 ± 0.2	10.4 ± 1.4

Groups of rats were selected for their level of inhibition in the Fixed Consecutive Number schedule of reinforcement task (FCN8); their level of activity in the Multiple Fixed interval/extinction schedules or their choice for the larger reward in the Delay discouting task. Scores measured in the 8-arm radial maze (mean of the 5 last sessions ± SEM) were compared between groups with low scores (LOW), intermediate (INT) and high scores (HIGH) in each task. Parameters measured in the radial maze (mean of the five last sessions ± SEM) were compared between groups. Statistical analysis of comparisons with HIGH, GLM with adjusted *p*-values (FDR procedure) **, *p *< .01, * *p *= .05.

## Discussion

This new experimental approach based on a differential analysis of distinct behaviours that may be related to impulsivity, demonstrates the relevance of the study of spontaneous individual differences in behaviour in rats and confirms the complexity of this trait. Large inter-individual differences in behaviours were identified and inhibitory control and hyperactivity were related to a higher sensitivity to psychostimulants and to working memory deficits respectively, two characteristics that are found in several impulsive-related psychopathologies.

### Individual differences in different aspects of impulsive-related behaviours

Marked individual differences were observed in all behavioural tasks. As previously reported [39,47], behavioural measures were stable and reproducible over the testing period. Impulsive rats in the fixed consecutive number schedule test had difficulty pressing first lever more than 6–7 times before switching to the other lever, thus making mainly inefficient chains of presses to obtain food. In contrast, the less impulsive rats made most of the responses efficiently, although a few rats exceeded the fixed ratio requirement (e.g., more than 15 presses independent of reward delivery). A few rats were excluded from the analysis because they failed to reach the criterion to be tested. This failure to meet criteria was probably due to impaired learning abilities rather than to being highly impulsive because the rats pressed the two levers at random and did not readily attain the FCN3 step. Of the rats that did reach criteria, non-impulsive rats did not differ from impulsive rats in the learning of the task, demonstrating that impulsive rats' lower efficiency was not due to a learning deficit. Furthermore, the rate of responding on both levers was higher in non-impulsive rats, probably because of their higher motivation for performing mainly rewarded chains of presses rather than hyperactivity, a finding that has been previously reported [39]. This behaviour cannot be attributed to a decrease in motivation for food reward, given that the latencies to collect food did not differ.

The two-component multiple FI-EXT schedule of reinforcement has been used to assess activity and impulsiveness in both laboratory and clinical populations, most notably in the Spontaneous Hyperactive Rat model of ADHD and in children diagnosed with ADHD [32]. The differences in activity levels between hyperactive *vs *hypoactive rats in both FI and EXT schedules were very important. They were about two-times higher than between Spontaneously Hypertensive strain or the Wistar Kyoto one in both schedules, even if the Sprague-Dawley strain is globally less active in this task compared to these two strains [32]. These differences in activity levels are remarkably similar to differences observed between ADHD children and their controls in a similar protocol [48].

This task assesses general behavioural inhibition in two different contexts. First, the reinforcement contingency during the fixed-interval schedule typically generates a "scalloped" pattern with little or no responding early in the interval and a progressive increase in rate when the opportunity for reinforcement delivery approaches [49]. Temporal discrimination is probably involved and excessive lever presses could reflect over-anticipation of the reward [50], perhaps caused by an over estimation of time. However, the hyperactivity observed in this task is not only related to earlier anticipation of reward, but also to higher sustained activity that lasts throughout the session. Second, during extinction, the absence of light (which had been paired with the availability of a reward during the fixed interval session) clearly indicates that no reinforcement is available. Thus, when the rat continues to press the lever excessively, this hyperactivity might be considered a perseverative behaviour, an aspect of disinhibition reflected by a tendency to pursue a goal-directed behaviour that is no longer appropriate and that might also be related to poor attentional processes [32]. Furthermore, a sensory discrimination deficit (i.e., perception of light changes between the fixed-interval and extinction sessions), can be excluded since all rats stopped responding at the beginning of an extinction schedule, indicating they perceived that the light was no longer present.

The delay-discounting task is the most widely used paradigm to test intolerance to situations when reward is delayed [43,51-53]. There were large inter-individual differences in rats in this task, similar to those observed using the T-maze delayed-discounting paradigm, and these differences, again, were independent of the ability to learn the task [54]. This characteristic also appears to be strain dependent; similar inter-individual differences have been reported between Sprague-Dawley and Spontaneously Hypertensive rat strains, while smaller differences have been found in the Wistar Kyoto strain [55].

### Relationship between different aspects of impulsive-related behaviours

Given that the different tasks were performed in the same apparatus, possible interactions between the tests should be considered. While learning the first task, the fixed consecutive number schedule, rats pressed mainly the left lever, while the right one was only used to terminate the chains of responses. For this reason, only the right lever was available in the following task (the multiple fixed-interval/extinction schedule of reinforcement). Mean performances of the whole group in both fixed-interval and extinction schedules were very similar to those described previously [32] and thus, it appears that an interaction with previous training had a negligible influence. The third procedure used (delay-discounting task) required both levers as in the fixed consecutive number schedule and thus, previous training might have elicited presses of the left lever. However, during the initial training in this task, presses on the left lever were associated with a small reinforcement whereas presses on the right one resulted in a large reinforcement with no delay. To ensure that previous training did not interact with the number of times the right lever was chosen, several sessions were performed until stable responding was reached. This training resulted in a stable percentage of choice for the right lever (large reinforcement) of 89%, a score similar to those previously reported in the literature in control rats [43,56,57].

Few inter-test relationships were found, strongly suggesting that the behaviours measured were not underpinned by a unitary process. This finding is consistent with data previously obtained in rats showing that motor impulsivity (measured with autoshaping procedure and conditioned locomotor activity to food) is independent of measures of impulsive choice and anticipated responding in a visual attention task [31]. It is worth noting that, a battery of tasks measuring different aspects of impulsivity in humans was used and no inter-correlation could be found confirming these results [58].

Numerous *intra-task *correlations were found and the more significant correlations obtained between the different tasks tested here concerned measures of similar activities attesting to the coherence of the measures. Specifically, these correlations concerned motor impulsivity during a waiting period before a reward, measured directly by lever presses during fixed-interval periods, and indirectly in the delay discounting task, through lever presses during time-out periods. Interestingly, activity during extinction periods was only correlated with total activity in the delay discounting task, confirming that this measure involves another aspect of motor impulsivity in which hyperactivity is observed independently of the context. The only significant relationship between different tasks *a priori *measuring different processes was observed between incapacity to terminate a chain of presses in the FCN8 task and hyperactivity during extinction periods. This relationship seems to be driven by rats that cannot extinguish their activity during extinction periods. These rats exhibit a high level of behavioural disinhibition that is reflected in the FCN8 task, namely they are less disposed to inhibit premature responding. This result is in line with the hypothesis that extinction deficit could explain response disinhibition [59].

### Relationship between impulsivity and locomotor responses

Sensation seeking and drug seeking are highly related to impulsiveness, and high novelty seekers are at increased risk for drug abuse [60]. A parallel between sensation-seeking in man and in animals has been established with novelty seeking behaviour which has been characterized with the use of several behavioural tasks assessing exploratory behaviour, behavioural response to different kinds of reinforcement like food or drugs (for review, see [36]). This trait in animals is well predicted by locomotor response to amphetamine as well as amphetamine self-administration [37]. Novelty and drug seeking behaviours both involve the mesolimbic dopaminergic system, in man and in rodents [36,60]. The task that best reveals the relationship between impulsivity and activity of this system is the FCN8 task since it showed that only impulsive rats in this task had a higher locomotor response to amphetamine. This task predominantly requires behavioural inhibition of premature responses that have negative consequences. In this context, anticipated responses could be considered as risk-taking behaviour. These behavioural responses are somehow reminiscent of aspects of the sensation-seeking trait in which risk-taking behaviour in many activities has been related [61,62]. Like sensation-seekers, LOW_FCN _rats may have deficits in estimating negative consequences of anticipated response: characteristics similar to those observed in the "impulsivity/sensation-seeking" trait described in humans.

In this study, the locomotor response to amphetamine is well correlated with response to novelty, as previously described [37]. However, response to novelty was not significantly related to impulsivity in the FCN8 task, probably because response to amphetamine amplifies inter-individual differences and thus magnifies differences between groups. This phenomenon has been similarly observed in previous experiments [38].

This study failed to show any relationship between impulsive-related behaviours and basal locomotor hyperactivity. Recent data obtained in our laboratory indicate that low environmental stimulation, in a confined place, is more suitable for revealing hyperactivity related to impulsiveness (unpublished data).

### Relationships between impulsivity and working memory

It has recently been proposed that deficits in the executive control system of working memory may explain some of the cognitive and behavioural problems exhibited by impulsive people [27,63,64]. Inversely, in a conceptual model of ADHD, it has been proposed that deficient inhibition could be the primary, bottom-up disturbance that impairs executive functions, such as working memory [20]. Only hyperactivity during an extinction schedule revealed an association with impaired working memory. This relationship favours the hypothesis of a cognitive deficit origin of the inability to extinguish a response [32]. However, it cannot be excluded that a deficient inhibitory response control like perseveration of a non-rewarded response, may explain both working memory errors and impaired extinction. Previous data, supporting this hypothesis, show that impulsive behaviour in the FCN8 task, which is related to the ability to inhibit responses during extinction (see second section of the discussion), has also been related to working memory deficits. This relationship was not observed in youth but during the aging process [39], indicating that the impulsivity trait may have long-term deleterious effects on cognitive functions.

## Conclusion

This new experimental approach based on inter-individual differences, describes a large variety of behaviours that may be related to impulsivity. This study, which is the first to systematically examine the behaviour of groups selected for their scores in several tasks, reveals the extent to which each aspect of impulsiveness and hyperactivity can be expressed in a normal population of rats and gives new insights on the meaning of these different aspects of adaptative behaviours. Further studies are needed to address other aspects of impulsive-related psychopathologies, but the strategy proposed here will help to define behavioural substrates required for animal models of impulsivity and to explore their neurobiological bases more accurately. Such an approach may ultimately help to broaden the understanding of psychiatric disorders that are characterized by a large variety of impulsive behaviours.

## Abbreviations

ADHD, attention deficit-hyperactivity disorder; CHX, delay-discounting task; EXT, extinction schedule; FCN, fixed consecutive number schedule; FI, fixed interval schedule; LOW, subgroup of rats with low scores; INT, subgroup of intermediate rats; HIGH, subgroup of rats with high scores; FDR, False Discovery Rate; ANOVA, analysis of variance; GLM, General Linear Model.

## Competing interests

The author(s) declare that they have no competing interests.

## References

[B1] Daruna JH, Barnes PA, McCown WG, Johnson JL, Shure MB (1993). The impulsive client: theory, research and treatment.. A neurodevelopmental view of impulsivity.

[B2] Evenden J (1999). Impulsivity: a discussion of clinical and experimental findings. J Psychopharmacol.

[B3] DSM-IV (1994). American Psychiatric Association, Committee on Nomenclature and Statistics: Diagnostic and Statistical Manual of Mental Disorders.

[B4] Moeller FG, Barratt ES, Dougherty DM, Schmitz JM, Swann AC (2001). Psychiatric aspects of impulsivity. Am J Psychiatry.

[B5] Rubia K (2002). The dynamic approach to neurodevelopmental psychiatric disorders: use of fMRI combined with neuropsychology to elucidate the dynamics of psychiatric disorders, exemplified in ADHD and schizophrenia. Behav Brain Res.

[B6] Taylor E (1998). Clinical foundations of hyperactivity research. Behav Brain Res.

[B7] Volkow ND, Fowler JS (2000). Addiction, a disease of compulsion and drive: involvement of the orbitofrontal cortex. Cereb Cortex.

[B8] Jentsch JD, Taylor JR (1999). Impulsivity resulting from frontostriatal dysfunction in drug abuse: implications for the control of behavior by reward-related stimuli. Psychopharmacology (Berl).

[B9] Disney ER, Elkins IJ, McGue M, Iacono WG (1999). Effects of ADHD, conduct disorder, and gender on substance use and abuse in adolescence. Am J Psychiatry.

[B10] Young SE, Mikulich SK, Goodwin MB, Hardy J, Martin CL, Zoccolillo MS, Crowley TJ (1995). Treated delinquent boys' substance use: onset, pattern, relationship to conduct and mood disorders. Drug Alcohol Depend.

[B11] Wilens TE (2004). Attention-deficit/hyperactivity disorder and the substance use disorders: the nature of the relationship, subtypes at risk, and treatment issues. Psychiatr Clin North Am.

[B12] Zuckerman M, Neeb M (1979). Sensation seeking and psychopathology. Psychiatry Res.

[B13] Zuckerman M (1993). P-impulsive sensation seeking and its behavioral, psychophysiological and biochemical correlates. Neuropsychobiology.

[B14] Lesieur HR, Rosenthal RJ (1991). Pathological gambling : a review of the literature. J Gambl Stud.

[B15] Pedinielli JL, Rouan G, Bertagne P (1997). Psychopathologie des addictions.

[B16] Baddeley AD (1986). Working memory.

[B17] Lenzenweger MF, Clarkin JF, Fertuck EA, Kernberg OF (2004). Executive neurocognitive functioning and neurobehavioral systems indicators in borderline personality disorder: a preliminary study. J Personal Disord.

[B18] Stevens A, Burkhardt M, Hautzinger M, Schwarz J, Unckel C (2004). Borderline personality disorder: impaired visual perception and working memory. Psychiatry Res.

[B19] Dinn WM, Harris CL, Aycicegi A, Greene PB, Kirkley SM, Reilly C (2004). Neurocognitive function in borderline personality disorder. Prog Neuropsychopharmacol Biol Psychiatry.

[B20] Barkley RA (1997). Behavioral inhibition, sustained attention, and executive functions: constructing a unifying theory of ADHD. Psychol Bull.

[B21] Adler CM, Holland SK, Schmithorst V, Tuchfarber MJ, Strakowski SM (2004). Changes in neuronal activation in patients with bipolar disorder during performance of a working memory task. Bipolar Disord.

[B22] Donaldson S, Goldstein LH, Landau S, Raymont V, Frangou S (2003). The Maudsley Bipolar Disorder Project: the effect of medication, family history, and duration of illness on IQ and memory in bipolar I disorder. J Clin Psychiatry.

[B23] Sweeney JA, Kmiec JA, Kupfer DJ (2000). Neuropsychologic impairments in bipolar and unipolar mood disorders on the CANTAB neurocognitive battery. Biol Psychiatry.

[B24] McGrath J, Chapple B, Wright M (2001). Working memory in schizophrenia and mania: correlation with symptoms during the acute and subacute phases. Acta Psychiatr Scand.

[B25] Peterson JB, Finn PR, Pihl RO (1992). Cognitive dysfunction and the inherited predisposition to alcoholism. J Stud Alcohol.

[B26] Finn PR (2002). Motivation, working memory, and decision making: a cognitive-motivational theory of personality vulnerability to alcoholism.. Behav Cogn Neurosci Rev.

[B27] Finn PR, Justus A, Mazas C, Steinmetz JE (1999). Working memory, executive processes and the effects of alcohol on Go/No-Go learning: testing a model of behavioral regulation and impulsivity. Psychopharmacology (Berl).

[B28] Deckel AW, Hesselbrock V (1996). Behavioral and cognitive measurements predict scores on the MAST: a 3-year prospective study. Alcohol Clin Exp Res.

[B29] Aytaclar S, Tarter RE, Kirisci L, Lu S (1999). Association between hyperactivity and executive cognitive functioning in childhood and substance use in early adolescence. J Am Acad Child Adolesc Psychiatry.

[B30] Evenden JL (1999). Varieties of impulsivity. Psychopharmacology (Berl).

[B31] Winstanley CA, Dalley JW, Theobald DE, Robbins TW (2004). Fractionating impulsivity: contrasting effects of central 5-HT depletion on different measures of impulsive behavior. Neuropsychopharmacology.

[B32] Sagvolden T (2000). Behavioral validation of the spontaneously hypertensive rat (SHR) as an animal model of attention-deficit/hyperactivity disorder (AD/HD). Neurosci Biobehav Rev.

[B33] Tripp G, Alsop B (2001). Sensitivity to reward delay in children with attention deficit hyperactivity disorder (ADHD). J Child Psychol Psychiatry.

[B34] McCourt WF, Gurrera RJ, Cutter HS (1993). Sensation seeking and novelty seeking. Are they the same?. J Nerv Ment Dis.

[B35] Dellu F, Mayo W, Piazza PV, Le Moal M, Simon H (1993). Individual differences in behavioral responses to novelty in rats. Possible relationship with the sensation-seeking trait in man. Pers Individ Dif.

[B36] Dellu F, Piazza PV, Mayo W, Le Moal M, Simon H (1996). Novelty-seeking in rats - Biobehavioral characteristics and possible relationship with the sensation-seeking trait in man. Neuropsychobiology.

[B37] Piazza PV, Deminiere JM, Le Moal M, Simon H (1989). Factors that predict individual vulnerability to amphetamine self-administration. Science.

[B38] Dellu-Hagedorn F (2005). Spontaneous individual differences in cognitive performances of young adult rats predict locomotor response to amphetamine. Neurobiol Learn Mem.

[B39] Dellu-Hagedorn F, Trunet S, Simon H (2004). Impulsivity in youth predicts early age-related cognitive deficits in rats. Neurobiol Aging.

[B40] Evenden JL (1998). The pharmacology of impulsive behaviour in rats II: the effects of amphetamine, haloperidol, imipramine, chlordiazepoxide and other drugs on fixed consecutive number schedules (FCN 8 and FCN 32). Psychopharmacology (Berl).

[B41] Berger DF, Sagvolden T (1998). Sex differences in operant discrimination behaviour in an animal model of attention-deficit hyperactivity disorder. Behav Brain Res.

[B42] Sagvolden T, Hendley ED, Knardahl S (1992). Behavior of hypertensive and hyperactive rat strains: hyperactivity is not unitarily determined. Physiol Behav.

[B43] Evenden JL, Ryan CN (1996). The pharmacology of impulsive behaviour in rats: the effects of drugs on response choice with varying delays of reinforcement. Psychopharmacology (Berl).

[B44] Adriani W, Laviola G (2003). Elevated levels of impulsivity and reduced place conditioning with d-amphetamine: two behavioral features of adolescence in mice. Behav Neurosci.

[B45] Olton DS, Samuelson RJ (1976). Remembrance of place passed: spatial memory in rats. J Exp Psychol.

[B46] Benjamini Y, Hochberg Y (1995). Controlling the False Discovery Rate: a practical and powerful approach to multiple testing. J R Stat Soc [Ser B].

[B47] Barratt ES, Patton JH, Zuckerman M (1983). Impulsivity: cognitive, behavioral and psychophysiological correlates. Biological bases of sensation-seeking, impulsivity, and anxiety.

[B48] Sagvolden T, Aase H, Zeiner P, Berger D (1998). Altered reinforcement mechanisms in attention-deficit/hyperactivity disorder. Behav Brain Res.

[B49] Ferster CB, Skinner BF (1957). Schedules of reinforcement.

[B50] Hata T, Okaichi H (2004). Medial prefrontal cortex and precision of temporal discrimination: a lesion, microinjection, and microdialysis study. Neurosci Res.

[B51] Bizot J, Le Bihan C, Puech AJ, Hamon M, Thiebot M (1999). Serotonin and tolerance to delay of reward in rats. Psychopharmacology (Berl).

[B52] Evenden JL, Ryan CN (1999). The pharmacology of impulsive behaviour in rats VI: the effects of ethanol and selective serotonergic drugs on response choice with varying delays of reinforcement. Psychopharmacology (Berl).

[B53] Thiebot MH, Le Bihan C, Soubrie P, Simon P (1985). Benzodiazepines reduce the tolerance to reward delay in rats. Psychopharmacology.

[B54] Poulos CX, Le AD, Parker JL (1995). Impulsivity predicts individual susceptibility to high levels of alcohol self-administration. Behav Pharmacol.

[B55] Adriani W, Caprioli A, Granstrem O, Carli M, Laviola G (2003). The spontaneously hypertensive-rat as an animal model of ADHD: evidence for impulsive and non-impulsive subpopulations. Neurosci Biobehav Rev.

[B56] Cardinal RN, Robbins TW, Everitt BJ (2000). The effects of d-amphetamine, chlordiazepoxide, alpha-flupenthixol and behavioural manipulations on choice of signalled and unsignalled delayed reinforcement in rats. Psychopharmacology (Berl).

[B57] Cardinal RN, Pennicott DR, Sugathapala CL, Robbins TW, Everitt BJ (2001). Impulsive choice induced in rats by lesions of the nucleus accumbens core. Science.

[B58] McDonald J, Schleifer L, Richards JB, de Wit H (2003). Effects of THC on behavioral measures of impulsivity in humans. Neuropsychopharmacology.

[B59] Johansen EB, Sagvolden T (2004). Response disinhibition may be explained as an extinction deficit in an animal model of attention-deficit/hyperactivity disorder (ADHD). Behav Brain Res.

[B60] Bardo MT, Donohew RL, Harrington NG (1996). Psychobiology of novelty seeking and drug seeking behavior. Behav Brain Res.

[B61] Zuckerman M, Lipsitt LP, Mitnick LL (1991). Sensation seeking: the balance between risk and reward. Self-regulatory behavior and risk-taking: causes and consequences.

[B62] Desrichard O, Denarie V (2005). Sensation seeking and negative affectivity as predictors of risky behaviors: a distinction between occasional versus frequent risk-taking. Addict Behav.

[B63] Villemarette-Pittman NR, Stanford MS, Greve KW (2003). Language and executive function in self-reported impulsive aggression. Pers Individ Dif.

[B64] Whitney P, Jameson T, Hinson JM (2004). Impulsiveness and executive control of working memory. Pers Individ Dif.

